# Over-the-counter antibiotic sales: a call for smarter stewardship rather than blanket restriction

**DOI:** 10.1080/20523211.2026.2709486

**Published:** 2026-09-07

**Authors:** Carl Llor

**Affiliations:** Institute for Primary Health Care Research Jordi Gol i Gurina (IDIAPJGol), Barcelona, Spain; CIBER de Enfermedades Infecciosas, Instituto de Salud Carlos III, Madrid, Spain; Research Unit for General Practice, Department of Public Health, University of Southern Denmark, Odense, Denmark

Dear Editor-in-Chief,

I read with great interest the study by Migiro et al. (2026) on the over-the-counter dispensing of antibiotics in community pharmacies in Tanzania. Despite legislation prohibiting this practice, non-prescription antibiotic dispensing remains widespread. The availability of over-the-counter antibiotics is particularly common in low- and middle-income countries, where regulations governing the sale and distribution of medicines are often weak or inadequately enforced, although non-prescription antibiotic dispensing also persists in some high-income countries, including parts of Europe (Auta et al., 2019; Guinovart et al., 2015). In many low- and middle-income countries with fragile healthcare systems, community pharmacies are often the first point of contact for individuals seeking healthcare. Understanding dispensing practices in these settings is therefore essential for addressing one of the major drivers of antimicrobial resistance.

Although the finding that nearly two-thirds of antibiotics were dispensed without a medical prescription is concerning, several additional results deserve particular attention. Most notably, more than one third of dispensed antibiotics belonged to the WHO AWaRe Watch and Reserve groups (World Health Organization, 2023), representing inappropriate use of agents that should be preserved because of their greater potential to select for antimicrobial resistance.

Equally concerning is the management of patients presenting with suspected urinary tract infections (UTIs), the most common indication for antibiotic dispensing in the study. Azithromycin and ciprofloxacin were the antibiotics most frequently dispensed. The authors suggest that azithromycin may have been preferred because its broad-spectrum activity makes it an attractive option when the diagnosis is uncertain. However, many patients presenting with UTIs have recurrent infections with characteristic symptoms, reducing diagnostic uncertainty. Furthermore, this prescribing pattern is difficult to justify in the Tanzanian context, where Gram-negative pathogens demonstrate low susceptibility to fluoroquinolones and third- and fourth-generation cephalosporins, with susceptibility rates of only approximately 20% (Hooft et al., 2026). Recent surveillance data also show that Escherichia coli exhibits approximately 95% resistance to azithromycin (Felician et al., 2026), whereas around 88% of urinary isolates remain susceptible to nitrofurantoin (Hooft et al., 2026). Nevertheless, nitrofurantoin, which is a first-line antibiotic in international guidelines (Bonkat et al., 2026), was dispensed to only 7% of patients with suspected UTIs.

These findings suggest that antibiotic selection in community pharmacies is frequently inconsistent with current treatment guidelines and local antimicrobial susceptibility patterns. Rather than focusing exclusively on eliminating all over-the-counter antibiotic sales – a challenging objective in many resource-limited settings – a more pragmatic and evidence-based initial strategy would be to prioritise restrictions on the non-prescription dispensing of Watch and Reserve antibiotics. Protecting these critically important agents should be a cornerstone of antimicrobial stewardship policies. At the same time, it is important to recognise that not all non-prescription antibiotic dispensing is necessarily inappropriate. In carefully selected situations, such as recurrent uncomplicated UTIs or recurrent genital herpes in patients who can reliably recognise their symptoms, limited non-prescription access may be clinically justifiable within an appropriate regulatory framework.

Reducing inappropriate antibiotic use requires comprehensive strategies that extend beyond regulation alone (Guinovart et al., 2018). Community pharmacists are ideally positioned to contribute to antimicrobial stewardship. Their role could evolve beyond dispensing medicines to include patient education, optimisation of antibiotic selection and treatment duration, point-of-care testing where available, and closer collaboration with prescribers to improve the quality of antibiotic use. Achieving this expanded role will require enhanced education in antimicrobial stewardship, continuous professional development, supportive regulatory frameworks, and stronger integration of pharmacists within primary healthcare teams (Bishop et al., 2019).

Finally, regulatory measures should also aim to minimise unnecessary antibiotic exposure. Restricting non-prescription access to selected Access-group antibiotics, ensuring the availability of smaller antibiotic pack sizes, and dispensing the exact number of tablets required to complete guideline-recommended short-course therapies could reduce unnecessary antibiotic consumption, particularly for self-limiting or likely viral infections, while promoting more rational antibiotic use and helping preserve antibiotic effectiveness for future generations.
